# Sero-prevalence of hepatitis B virus markers and associated factors among children in Hawassa City, southern Ethiopia

**DOI:** 10.1186/s12879-020-05229-7

**Published:** 2020-07-22

**Authors:** Bedru Argaw, Adane Mihret, Abraham Aseffa, Azeb Tarekegne, Siraj Hussen, Demelash Wachamo, Techalew Shimelis, Rawleigh Howe

**Affiliations:** 1 Department of Medical Laboratory Science, Hawassa College of Health Sciences, South Nations and Nationalities Peoples Region, Hawassa, Ethiopia; 2 Armauer Hansen Research Institute, Addis Ababa, Ethiopia; 3 School of Medical Laboratory Science, College of Medicine and Health Sciences, Hawassa University, Hawassa, Ethiopia; 4 Department of Public Health, Hawassa College of Health Sciences, South Nations and Nationalities Peoples’ Region, Hawassa, Ethiopia

**Keywords:** Hepatitis B virus, Sero-prevalence, Hawassa, Southern Ethiopia

## Abstract

**Background:**

Hepatitis B virus (HBV) infection is one of the major public health problems worldwide. Limited information exists about the epidemiology of HBV infection in Ethiopia. This study aimed to assess sero-prevalence of HBV markers and associated factors in children living in Hawassa City, southern Ethiopia.

**Methods:**

A community-based cross-sectional study was conducted among 471 children in Hawassa City, southern Ethiopia from May to September, 2018. A total of 471 children were included in the study using a multistage sampling technique. Data on demographic and risk factors were gathered using structured questionnaires. Blood samples were collected and sera were screened for hepatitis B surface antigen (HBsAg), antibody to core antigen (anti-HBc), and antibody against surface antigen (anti-HBs) using enzyme-linked immunosorbent assay.

**Results:**

The sero-prevalence of HBsAg, anti-HBc, and anti-HBs markers among children were 4.4, 19.5 and 20.0%, respectively. Children at higher risk of having HBsAg marker were those who had a history of injectable medications (AOR 5.02, 95% CI: 1.14, 22.07), a family history of liver disease (AOR 6.37, 95% CI: 1.32, 30.74), a HBsAg seropositive mothers, (AOR 11.19, (95% CI: 3.15, 39.67), and had no vaccination history for HBV (AOR, 6.37, 95% CI: 1.32, 30.74). Children from families with low monthly income, who were home delivered, unvaccinated for HBV or with HBsAg seropositive mother had increased risk of having anti-HBc.

**Conclusions:**

The study findings showed an intermediate endemicity of HBV infection in the study setting. The observed rate of residual HBV infection with low rate of immunized children after HBV vaccination was high. Hence, introducing birth dose vaccine, safe injection practice and improving immunization coverage during pregnancy as part of the antenatal care package should be considered. Furthermore, governmental and non-governmental organizations should give attention on timely measures for the prevention of ongoing vertical transmission from mother to child as well as early horizontal transmission of HBV in Hawassa City, Ethiopia.

## Background

Hepatitis B virus (HBV) is a deoxyribonucleic acid (DNA) virus classified under the hepadnaviridae family [1, 2]. About 257 million people globally had chronic HBV infection in 2015 [3, 4]. It was estimated that, annually, about 4.5 million new cases occurred, and 887, 000 people die globally from chronic sequelae of HBV infection including cirrhosis (52%) and liver cancer (38%) [5]. Africa has the second largest number of chronic HBV carriers after Asia and is considered as a region of highly endemicity [6]. About 60 million people in Africa are chronically infected with HBV and an estimated prevalence of 6.1% in adult population [7, 8].

HBV transmission occurs through sexual exposure, transfusion of infected blood, and use of unsterilized equipment for medical procedures and sharing of sharp materials [9]. However, perinatal exposure to HBV is the most common mode of transmission in areas of medium to high endemicity [10]. Babies born to a mother who is positive for HBsAg and HBeAg markers have ≥90% chance of contracting the infection and becoming chronic carrier [11]. Of these children, 15 to 25% have risk of dying from cirrhosis or liver cancer during adulthood [12, 13].

HBV is an important public health problem in Ethiopia, and the epidemiology varies with geographical area, population practice, age and mode of acquisition [10, 14, 15]. A previous national survey showed that 10.8% of young men had HBsAg, and 73.3% had at least one HBV marker [16]. A 7% sero-prevalence of HBsAg was also reported in a community-based study conducted in Addis Ababa [10].

The World Health Organization (WHO) set a strategy to reduce HBV incidence in children under-five to below 1% by 2020, and decreasing the prevalence to 0.1% by 2030 [3]. In Ethiopia, HBV vaccination has been introduced to the national Expanded Program for Immunization (EPI) since 2007 [17]. The current immunization schedule for children under one year of age in the country includes; BCG, measles, DPT-HepB-Hib or penta-valent vaccine, OPV. The HBV vaccination as (DPT3/PENTA 3) coverage was 73 and 86% in 2007 and 2011, respectively in Ethiopia [18]. To assess the progress of interventions, understanding the epidemiology of HBV infection and vaccination status would be important. However, there have been limited data on HBV infection in Ethiopia. Therefore, this study aimed to determine sero-prevalence of HBV markers (HBsAg, anti-HBc and anti-HBs) among children at Hawassa City, Southern Ethiopia.

## Methods

The study was conducted in Hawassa City in Southern Ethiopia. Hawassa City has 8 sub-cities and located 272 km south of Addis Ababa, Ethiopia (Hawassa city administration, 2011). The city administration is divided into 7 urban sub-cities, containing 20 *kebeles* (smallest administrative unit) and one rural sub-city with 12 *kebeles*. According to the report of housing and population census (CSA, 2009), the population size of Hawassa City Administration in 2018 was 374,034; of which 190,757 were males and 183,277 were females [19]. The total children aged 5–8 years old were 56,252 and the total number of households were 78,124.

A community based cross-sectional study was conducted from May to September, 2018. The study population consisted of children aged 5–8 years and who were found at home during the study period. Mothers and/or children who were sick or on antiretroviral therapy or unwilling to participate in the study were excluded. Further, children who had not received full dose vaccine were considered to have no history of vaccination and were excluded from the study.

The sample size was determined using a single population proportion formula with assumptions of 5.3% HBsAg sero-prevalence among children [20], 95% confidence interval, and 3% marginal of error (d). The sample size calculated was 471 after considering a 10% non-response rate and design effect of 2.

$$ \mathrm{n}={\left({Z}_{\raisebox{1ex}{$1-a$}\!\left/ \!\raisebox{-1ex}{$2$}\right.}\right)}^2\frac{P\left[1-P\right]}{d^2}, $$

Where.

**n** = Sample size.

**Z** = Standard normal distribution value at the 95% CI, which is 1.96.

**P** = The prevalence of HBV infections 5.3% and.

**D** = The margin of error, taken as 0.03%. Hence.

$\mathrm{n}=\frac{(1.96)^2(0.053)(0.953)}{(0.30)^2}=214$**. The final sample size was adjusted as follows:**

Sample size = n (sample size) + (10% non-respondent) X Design effect (2). Thus, final sample size (n) was calculated as n = (214+ 21.4) X 2 = 470.85 ~ **471.**

A multistage random sampling technique was used. Two sub-cities were selected using a simple random sampling technique in the first stage, and four *kebeles* were selected in the second stage. In the third stage, the total sample size was proportionated to the four *kebeles*. In the last stage, households of eligible study participants were selected using systematic random sampling technique, and study participants were selected using lottery method (Fig. S1).

Pre-tested and structured questionnaires were used to collect information on socio-demographic characteristics and other associated factors. The vaccination status of children was collected from immunization cards and/or by asking mothers. Health workers conducted face-to-face interviews with mothers and gathered the data. Training on data collection and sample drawing and transportation were given, and pretest was done to validate the questionnaire prior to actual work.

### Serological analysis

About 5 ml of blood sample was drawn from every child and mother. Samples were transported within 6 h of collection to the laboratory of the Hawassa University Comprehensive Specialized Hospital using cold box. Separated sera were stored at -80 °C and then transported to the Armauer Hansen Research Institute (AHRI) in Addis Ababa for analyses. Sera were tested for HBsAg, anti-HBc, and anti-HBs using enzyme-linked immunosorbent assay (ELISA) (Monolisa PLUS, BIO-RAD, France). Testing was performed according to the instructions of the manufacturer.

### Data quality assurance

The questionnaire was prepared in English language and translated to Amharic language and then back to English. One week prior to data collection, the questionnaire was pre-tested on 5% of the calculated sample size at Adare Hospital other than the actual study sites to ensure questions were unambiguous. Prior to the beginning of data collection, all data collectors were trained by the principal investigator. The collected data were checked daily for consistency and accuracy. Standardized procedures were strictly followed during sample collection, storage and analytical process. The quality of test results was maintained using known negative and positive samples as external quality controls.

### Operational definitions

- HBV infected: whose blood is serologically positive for HBsAg
- HBV immune following a resolved infection: whose blood is serologically HBsAg negative, anti-HBc positive and anti-HBs positive
- HBV immune following vaccination: anti-HBs positive after vaccination with anti-HBs titer ≥10mIU/ml.
- HBV susceptible: HBsAg negative, anti-HBc negative and anti-HBs negative

### Data analysis

Data entry, cleaning and analysis was done using SPSS version 23.0 software. Frequencies and percentages were calculated to summarize results of categorical variables. Bivariate logistic regression analysis was conducted to compute crude odds ratio (COR). Variables with a *p*-value < 0.25 in bivariate analysis were candidates for multivariable logistic regression. Adjusted odds ratios (AOR) with 95% confidence intervals (CI) were used to measure strength of the association between HBV infection and its determinant factors. A p-value < 0.05 was considered as a significant association.

## Results

### Socio-demographic characteristics

A total of 471 study participant were enrolled, of which, 451 (95.8%) were included in the analysis. The mean age of the children was 6.56 years (standard deviation SD), 1.22; range, 5–8 years), and 232 (51.4%) were boys. Among the study participants, 147 (32.6%) were from Alamura, 119 (26.4%) from Hogane-Wacho, and the remaining were from Dume and Wukiro *kebeles*. (Table 2).

### Sero-prevalence of hepatitis B virus markers (HBsAg, anti-HBc and anti-HBs)

The overall sero-prevalence of HBsAg, anti-HBc, and anti-HBs among children was 4.4% [95% confidence interval (CI): 2.8–6.6], 19.5% [95% CI: 16.1–23.4] and 20.0% [95% CI: 16.5–23.8], respectively. All children with HBsAg were also positive for anti-HBc, and 37 (8.4%) participants were positive for both anti-HBs and anti-HBc markers. Of the children, 53 (11.6%) had HBV immunity following vaccination (anti-HBs+) (Table 1). In addition, the prevalence of HBsAg among paired mothers was 7.1% [95% confidence interval (CI): 4.7–9.8] (Table 2).

**Table 1:** Sero-prevalence of hepatitis B virus markers among children at Hawassa City, southern Ethiopia in 2018. (*n* = 451)

Hepatitis B virus sero-markers	No.	%	95.0% CI
HBsAg (+), Anti-HBs (−), and Anti-HBc (+)	20	4.4	2.7–6.4
Anti-HBc +	88	19.5	15.7–23.3
Anti-HBs +	90	20.0	16.5–23.8
Anti-HBs (+), Anti-HBc (+), HBsAg (−)	37	8.4	6.0–11.1

**Table 2:** Bivariate and multivariable analysis of sociodemographic and self reported associated factors for HBsAg positivity among children at Hawassa City, Southern Ethiopia in 2018

Variables	Serostatus of HBsAg of children						
	Total		Positive				
	No.	(%)	No.	(%)	COR (95% CI)	AOR (95%CI)	P-Value
Age in years							
5 & 6	226	(50.1)	13	(5.8)	1.90 (0.74, 4.86)	2.39 (0.73, 7.88)	0.151
7 & 8	225	(49.9)	7	(3.1)	1	1	
History of day care							
No	306	(67.8)	8	(2.6)	1	1	
Yes	145	(32.2)	12	(8.3)	3.36 (1.34, 8.41)	2.83 (0.95, 8.47)	0.062
Average monthly income							
> 2000 ETB	296	(65.6)	10	(3.4)	1	1	
≤ 2000 ETB	155	(34.4)	10	(6.5)	1.97 (0.80, 4.85)	2.02 (0.64, 6.37)	0.231
History of hospital admission							
No	419	(92.9)	16	(3.8)	1	1	
Yes	32	(7.1)	4	(12.5)	3.60 (1.13, 11.49)	1.48 (0.27, 8.04)	0.652
History of injectable medications							
No	415	(92.0)	12	(2.9)	1	1	
Yes	36	(8.0)	8	(22.2)	9.60 (3.63, 25.39)	5.02 (1.14, 22.07)	0.033*
Place of birth							
Health facility	270	(59.9)	9	(3.3)	1	1	
Home	181	(40.1)	11	(6.1)	1.88 (0.76,4.62)	1.65 (0.49, 5.59)	0.422
History of vaccination							
No	215	(47.7)	15	(7.0)	3.47 (1.24,9.70)	6.37 (1.32,30.74)	0.021*
Yes	236	(52.3)	5	(2.1)	1	1	
Family history of liver disease							
No	425	(94.2)	15	(3.5)	1	1	
Yes	26	(5.8)	5	(19.2)	6.51 (2.16, 19.61)	6.37 (1.32, 30.74)	0.021*
Sero-status of mother for HBsAg							
Negative	419	(92.9)	12	(2.9)	1	1	
Positive	32	(7.1)	8	(25.0)	11.31 (4.22, 30.27)	11.19 (3.15, 39.67)	< 0.001**

**NB:** *Candidate variable for multivariate analysis at *P* < 0.25 *variable significant and ^**^variable highly significant by the multivariate analysis at *P* < 0.05 **COR:** crude odds ratio, **AOR:** adjusted odds ratio, **CI:** confidence interval, **P-V**: p -value, **1:** reference.

### Associated factors for hepatitis B virus infection among children

Of the study participants, 32 (7.1%) had a history of hospital admission and 36 (8%) had history of injectable medications. The number of children born at home was181 (40.1%), and 215 (47.7%) children were not vaccinated (Table 2).

In multivariable analysis, children who had history of injectable medications were 5 times (AOR 5.02: 95% CI: 1.14, 22.1) more likely to have HBV infection. Children who had a family history of liver disease were 6 times more likely to be exposed to HBV infection (AOR 6.37, 95% CI: 1.32, 30.7). In addition, children born to mothers with HBsAg were at higher risk of HBV infection than children whose mothers were negative for HBsAg (AOR 11.2, (95% CI: 3.15, 39.67). Moreover, children who had no history of vaccination were 6 times more likely to have HBV infection (AOR, 6.36, 95% CI: 1.32, 30.74) as compared to their counterparts (Table 2).

### Associated factors for hepatitis B virus exposure among children

After further analysis for those significantly associated variables in multivariable logistic regression analysis, children with family monthly income < 2000 ETB (AOR 2.15, 95% CI:1.25, 3.72), home delivered (AOR 2.82, 95% CI:1.58, 5.06), a history of vaccination (AOR 2.45, 95% CI:1.41, 4.27) or with a HBsAg sero-positive mother (AOR 2.59, 95% CI: 1.13, 5.96) had higher sero-positivity for anti-HBc compared to their counterparts. There was no statistically significant association between self-reported risk factors including average monthly income, history of hospital admission, age and history of day care with HBV infection (Table 3).

**Table 3:** Bivariate and multivariable analysis of sociodemographic and self-reported associated factors for anti-HBc positivity among children at Hawassa City, Southern Ethiopia, 2018

	Anti-HBc of children						
**Variables**	**Total**		**Positive**				
	**No.**	**(%)**	**No.**	**(%)**	**COR (95% CI)**	**AOR (95% CI)**	**P-Value**
Age in years							
5 & 6	226	(50.1)	32	(14.2)	1	1	
7 & 8	225	(49.9)	56	(24.9)	2.01 (1.24, 3.25)	1.46 (0.80, 2.66)	0.221
History of day care							
No	306	(67.8)	58	(19.0)	1		
Yes	145	(32.2)	30	(20.7)	1.12 (0.68, 1.83)		0.664
Average monthly income							
> 2000 ETB	296	(65.6)	46	(15.5)	1	1	
≤ 2000 ETB	155	(34.4)	42	(27.1)	2.02 (1.26, 3.24)	2.15 (1.25, 3.72)	0.006*
History of hospital admission							
No	419	(92.9)	79	(18.9)	1	1	
Yes	32	(7.1)	9	(28.1)	1.68 (0.75, 3.78)	1.14 (0.38, 3.45)	0.593
History of injectable medications							
No	415	(92.0)	78	(18.8)	1	1	
Yes	36	(8.0)	10	(27.8)	1.66 (0.77, 3.59)	1.33 (0.46, 3.82)	0.593
Place of birth							
Health facility	270	(59.9)	27	(10.0)	1	1	
Home	181	(40.1)	61	(33.7)	4.58 (2.77, 7.57)	2.82 (1.58, 5.06)	< 0.001*
History of vaccination							
No	215	(47.7)	63	(29.3)	3.50 (2.11, 5.81)	2.45 (1.41, 4.27)	0.002*
Yes	236	(52.3)	25	(10.6)	1	1	
Family history of liver disease							
No	425	(94.2)	82	(19.3)	1		
Yes	26	(5.8)	6	(23.1)	1.26 (0.49, 3.22)		0.637
Sero-status of mother for HBsAg							
Negative	419	(92.9)	76	(18.1)	1	1	
Positive	32	(7.1)	12	(37.5)	2.71 (1.27, 5.78)	2.59 (1.13, 5.96)	0.025*

## Discussion

To reduce the morbidity and mortality related to HBV infection, WHO recommended that all countries should introduce the vaccine in routine immunization programs by 1995 [21]. Ethiopia introduced HBV vaccine to the national EPI program in 2007. However, no data are available on epidemiology of HBV infection in children born after the introduction of the vaccine. We assessed the sero-prevalence of HBV markers among children in Hawassa City, Ethiopia.

The sero-prevalence of HBsAg, anti-HBc, and anti-HBs positivity among children aged 5–8 years was 4.4% [95% CI: 2.7–6.4], 19.5% [95% CI: 16.1–23.4] and 20.0% [95% CI: 16.5–23.8], respectively. According to the criteria of WHO, the observed prevalence of HBsAg showed an intermediate endemicity of HBV infection in the study area [22]. The prevalence of HBsAg in this study was in agreement with results reported in similar study population in Gambia (2.8%) [23]. However, a higher prevalence was reported in Ghanaian rural children (21%) [24], and a lower prevalence was reported from Senegal (2.0%) [25] and Lao People’s Democratic Republic (2.1%) [26]. Further, the prevalence of anti-HBc in this study was lower than results from Gambia (31%) [23], Senegal (27%) [25] and Ghana (75%) [24]. A higher prevalence of anti-HBs was reported in studies conducted in Egypt (57.7–67%) [27] and Senegal (58%) [25]. The difference in prevalence of HBsAg, anti-HBc and anti HBs might be due to variability of study methods employed and diverse risk factors involved in various geographical regions.

Children who had history of using injectable medications were more likely to have HBV infection. Even though direct comparison is difficult because of difference in study population, consistent finding was reported in China [28] and Saudi Arabia [29]. This may be due to proper usage of universal precautions, sharing needle and using unsterilized equipment’s for medical purpose, which increase the risk of infection. Home delivered children had high seropositivity of anti-HBc, as also reported in studies conducted in Addis Ababa, Ethiopia [10] and Saudi Arabia [29]. This may be due to the use of unsterilized or inadequately sterilized instruments. A higher risk of HBV infection in children from family with a history of liver disease compared with those with no family history of liver disease. This result was similar with the study conducted in China [28], Saudi Arabia [29] and USA [30]. This may be due to increased chance of coming in contact with an infected person’s blood and other body fluids.

Children with history of vaccination had lower risk of having HBV infection compared to their counterparts. This result was similar with the study conducted in Papua New Guinea [31], Uganda [32], Pakistan [33] and Nigeria [34]. The study result emphasizes the importance of receiving full HBV vaccination to protect against the infection. It was also observed that the prevalence of residual HBV infection was higher among children with lower rate of immunization (53.4%). According to the Ethiopian demographic health survey report, the coverage of DPT-HepB-Hib3 and all vaccine in urban area of Ethiopia were 60.5, and 48.1%, respectively [35]. This may be explained by absence of HBV birth dose and low maternal education. This study revealed that children who were not vaccinated showed a higher sero-positivity of anti-HBc compared to their counter parts. Similar findings were reported from Uganda [32] and Nigeria [34], indicating a higher risk of exposure to HBV infection among non-vaccinated children.

Maternal HBsAg seropositivity also increased the risk of HBV infection among children. This result was similar with the study conducted in Pakistan [33], Taiwan [36] and Iran [37]. This may be due to a higher risk of HBV transmissibility from infected mother to child during birth, intrauterine transmission in HBeAg-positive mothers with high HBV viral load (> 200,000 IU/ml), and horizontal transmission through sharing sharp materials (like razor blade, and needle) and body fluid exposure [38, 39]. The observed association between mothers HBsAg positivity and child anti-HBc positivity was also shown in studies in Pakistan [33], Taiwan [36] and Iran [37]. This may be due to the fact that HBV infected mothers transmit the virus to their child vertically during birth or horizontally afterwards. Children from family with low monthly income had a higher prevalence of anti-HBc, which was consistent with results from low and middle income countries [40]. In relation to economic situations, an increased risk of exposure to share sharp materials and having lower health care utilization might occur.

Our study had some limitations. First, we did not include testing for HBeAg, anti-HBe and HBV viral load, which are also important determinants of the HBV transmission. Second, the study was done in urban populations and may underestimate the true burden of the disease in the rural community. Third, we did not screen children for HIV infection, which might be considered as missed opportunity to assess the influence of HIV on response to HBV vaccine. Despite these shortcomings, this study provides relevant epidemiological information about HBV infection in children in the study area.

## Conclusions

The study findings showed an intermediate endemicity of HBV infection in the study setting. Histories of injectable medication, family liver disease, lack of vaccination and maternal HBsAg sero-positivity were independent predictors of HBV infection. The observed rates of residual HBV infection with low rate of immunized children after HBV vaccination was high. Therefore, introducing hepatitis B vaccine and possibly hepatitis B immunoglobulin within 12 h of birth for infants born to infected mothers and provision of treatment for high viremic pregnant mothers, as well as safe injection practice would be important interventions. Furthermore, governmental and non-governmental organizations should give attention to timely measures for the prevention of ongoing vertical transmission from mother to child as well as early horizontal transmission of HBV in Hawassa/Ethiopia.

## Supplementary information

**Additional file 1: Fig. S1.** The sampling technique from Hawassa city, Southern Ethiopia, 2019.

## Data Availability

There is no remaining data and materials, all information is clearly presented in themain manuscript.

## Abbreviations

HBV: Hepatitis B virus
HBsAg: Hepatitis B surface antigen
Anti-HBc: Antibody to core antigen
Anti-HBs: Antibodies against surface antigen
HBIG: Hepatitis B immunoglobulin
