# Supplementary material for: Sero-prevalence of hepatitis B virus markers and associated factors among children in Hawassa City, southern Ethiopia
Source: BMC Infect Dis. 2020 Jul 22;20:528. doi: 10.1186/s12879-020-05229-7 (PMC7374845; doi:10.1186/s12879-020-05229-7)
Supplement: Supplementary file 1 — Additional file 1: Fig. S1. The sampling technique from Hawassa city, Southern Ethiopia, 2019. [file 12879_2020_5229_MOESM1_ESM.docx]

**Systematic random sampling**

**Proportional sampling**

**Systematic random sampling**

**Systematic random sampling**

Figure S1: The sampling technique from Hawassa city, Southern Ethiopia, 2019.
